# Analysis of surgical and oncological outcome in internal and external hemipelvectomy in 34 patients above the age of 65 years at a mean follow-up of 56 months

**DOI:** 10.1186/s12891-015-0494-5

**Published:** 2015-02-18

**Authors:** Wiebke K Guder, Jendrik Hardes, Georg Gosheger, Marcel-Philipp Henrichs, Markus Nottrott, Arne Streitbürger

**Affiliations:** Department of Orthopedics and Tumor Orthopedics, University Hospital Muenster, Albert-Schweitzer-Campus 1, Building A1, 48149 Muenster, Germany

**Keywords:** Hemipelvectomy, Hindquarter amputation, Elderly patients

## Abstract

**Background:**

With an increasing life expectancy and improved treatment regimens for primary or secondary malignant diseases of soft tissue or bone, hemipelvectomy will have to be considered more often in elderly patients in the future. Scientific reviews concerned with the surgical and oncological outcome of elderly patients undergoing hemipelvectomy are scarce. Therefore, it is the purpose of this study to review the outcome of patients treated with that procedure at our hospital and investigate the feasibility of such extensive procedures at an increased age.

**Methods:**

A retrospective analysis of thirty-four patients who underwent hemipelvectomy at an age of 65 years or older was performed to determine their surgical and oncological outcome. The Kaplan-Meier method was used to calculate the cumulative probability of survival using the day of tumor resection as a starting point. Univariate analysis was carried out to investigate the influence of a particular single parameter.

**Results:**

The mean age at operation was 70.2 years. Thirty patients were treated for intermediate- to high-grade sarcoma and 81.8% of tumors were larger than or equal to 10 cm in the longest diameter. Thirteen patients underwent internal hemipelvectomy and nine patients external hemipelvectomy as a primary procedure. Twelve patients were treated with external hemipelvectomy after failed local tumor control at primary operation. Wound infection occurred in 61.7% of cases. Three patients underwent amputation for non-manageable infection after internal hemipelvectomy. Hospital mortality was 8.8%. Clear resection margins were obtained in 88% of patients; in another 6% of patients planned intralesional resections were performed. Local recurrence occurred in 8.8% of patients at a mean time of 26 months after operation. Eleven patients are alive with no evidence of disease and 23 patients died of disease or other causes. Patients with pulmonary metastases had a mean survival period after operation to DOD of 22 months compared to 37 months in the curative group.

**Conclusion:**

Despite an elevated rate in hospital mortality and wound infection, this study suggests that hemipelvectomy is feasible in elderly patients, although requiring long hospitalization periods and causing a limited functional outcome.

## Background

Pelvic surgery is an accepted treatment for primary malignant tumors of soft tissue and bone. Depending on tumor size, site, proximity to neurovascular structures and abdominal organs as well as the stage of disease, the indication for carrying out an internal or external hemipelvectomy has to be evaluated.

Internal hemipelvectomy is a surgical procedure of partial to complete unilateral resection of bone and soft tissue of the pelvis with preservation of the adjacent leg, whereas in external hemipelvectomy - also referred to as hindquarter amputation - the adjacent leg is resected as well. Internal hemipelvectomy is indicated when tumor resection with wide margins can be obtained without sacrificing the neurovascular structures and the remaining tissue (muscle, subcutaneous tissue and skin) is acceptable to perform a functional reconstruction. External hemipelvectomy is indicated when the neurovascular structures are compromised by tumor growth, as well as in recurrent tumors when clear surgical margins can only be obtained by performing ablative procedures.

The overall prognosis of pelvic tumors is still inferior compared to the results achieved in extremity surgery for different tumor entities [1-3]. Complications after operations occur frequently, including genitourinary and gastrointestinal injuries, mass bleeding, infection or vascular occlusion [4,5].

A review of recent literature reveals that hemipelvectomies are performed in all age groups with predominance in younger patients with a mean or median age ranging between 17.9 and 60 years [3,6,7]. The number of patients treated with a hemipelvectomy above the age of 65 years per study is small and Ham et al. show that performing the procedure in older patients has not always been deemed feasible in the past [8,9].

But due to an improvement of oncological therapies, internal and external hemipelvectomy also come into focus as a means of treating metastatic disease in addition to soft tissue and bone sarcomas of the pelvis [6,10]. Therefore, the feasibility of hemipelvectomy in older patients with comorbidities as an aggravating factor will need to be assessed more frequently in the future.

With an increasing significance of major pelvic surgery as a treatment option for elderly patients and a lack of existing studies on this subgroup’s overall outcome, we retrospectively evaluated the surgical and oncological outcome in patients above the age of sixty-five years who have been treated with hemipelvectomy at our department from 1999 until 2012. Therefore, the aim of this study is to analyze whether these patients benefit from hemipelvectomy in terms of surgical safety and oncological follow-up.

## Methods

### Patient eligibility

Records of patients who underwent internal and external hemipelvectomy at our department between 1999 and 2012 were retrospectively reviewed.

Thirty-four patients who were sixty-five years of age or older at the time of operation and underwent hemipelvectomy for primary bone or soft tissue sarcomas, bony metastases or benign bony lesions in the pelvic region were considered eligible for the analysis. Patients were followed up quarterly in our outpatient clinic for the first two years after operation. Magnetic resonance imaging (MRI) and x-rays of the pelvis were performed for local follow-up. Computed tomography (CT) scans and x-rays of the chest were obtained biannually, each procedure once per year. Until completion of the fifth year after surgery biannual follow-ups were scheduled. Patients, family members, attending physicians and the local residents’ registration office were contacted for information on the current state of disease when patients didn’t follow up in our outpatient clinic as recommended.

### Diagnostic staging

Conventional x-rays and magnetic resonance imaging were mandatory for depiction of the pelvic tumor prior to operation. A CT of the chest and a 99-Tc-methylene diphosphonate bone scan were also required. The stage of disease at the time of operation was defined according to the system of the Musculoskeletal Tumor Society [11]. Pelvic resections were classified using the system of Enneking and Dunham [12]. Measurement of the largest tumor diameter was performed on the MR images or taken from histopathological reports, sorting them into three groups (small: ≤ 10cm, intermediate: ≥ 10 cm, large: ≥ 20cm).

Except for selected cases, operation aimed at a complete removal of the tumor with wide or radical margins. Surgical and histopathological reports were used to define the margins according to Enneking [13]. Local failure was defined as development of a local recurrence or tumor progression after intralesional operation.

### Patient characteristics

Out of thirty-four patients, twenty patients were male (59%) and fourteen patients female (41%). The mean age at operation was 70.2 years (range 65 – 83 years). The mean body mass index (BMI) was 26.2 (range 20.8 – 37.7). Severe comorbidities were known in 47% of patients (i.e. cardiovascular disease, diabetes mellitus, stroke, second neoplastic disease).

In twenty-three cases (68%) the tumor originated in the bone; eleven cases (32%) of soft tissue sarcoma were treated. Histological subtypes can be seen in Table 1. In two cases a dedifferentiation of low-grade chondrosarcoma in recurrent/persistent tumors was observed. The main tumor mass was located in the ilium in thirteen cases (38.2%), the proximal thigh in seven cases (20.6%), the acetabulum in five cases (14.7%), the gluteal region in four cases (11.8%), the proximal femur in three cases (8.8%) and the ischiopubic bone and inguinal region in one case (2.9%) each. The largest tumor diameter was ≤ 10 cm in 18.2% of cases (n = 6), ≥ 10 cm in 54.5% of cases (n = 18) and ≥ 20 cm in 27.3% of cases (n = 9). In one patient, information on tumor size was not available. The stage of disease was as follows in decreasing order: IIB (high-grade extracompartimental) n = 19, IIIB (metastatic extracompartimental) n = 8, IB (low-grade extracompartimental) n = 4 and IIA (high-grade intracompartimental) n = 1. In one case, a benign Stage III lesion (locally aggressive) was found. In one case the staging system didn’t apply (#31 in Tables 2 and 3). An overview of treated cases can be seen in Tables 2 and 3. Twenty-five patients were treated with a curative intent whereas hemipelvectomy was carried out in nine patients with a palliative intention. A curative intention was defined as the ability to achieve clear surgical margins in patients who showed no metastases at the time of operation.

**Table 1:** **Frequency distribution of diagnosis and grading in patients treated with hemipelvectomy**

**Diagnosis**	**Bone tumor**						**Soft tissue tumor**		
**Grading**	**Benign**	**Regressive**	**Low-grade (G1)**	**Intermediate-grade (G2)**	**High-grade (G3)**	**Total**	**Intermediate-grade (G2)**	**High-grade (G3)**	**Total**
**Chondrosarcoma**			2	9	7	**18**			
**Pleomorphic sarcoma**					1	**1**	1	6	**7**
**Leiomyosarcoma**							2	1	**3**
**Osteosarcoma**					2	**2**			
**Fibrosarcoma**								1	**1**
**Metastasis thyroid cancer**		1				**1**			
**Giant cell tumor**	1					**1**			
**Total**	**1**	**1**	**2**	**9**	**10**	**23**	**3**	**8**	**11**

**Table 2:** **Overview of patient characteristics, surgical outcome**

**Case**	**Age at operation**	**Tumor entity**	**Grading**	**Tumor site**	**Tumor size (cm)**	**Tumor stage**	**Operation indication**	**Type of procedure**	**Subtype of procedure**	**Surgical revision (n)**	**VAC duration Y/N days**
1	68	GCT	benign	ischiopubic	6x6x7	III (benign)	PR	int intra	P3	N	N
2	69	LMS	2	prox thigh	19	IIB	SR LR	ext	P2/3	1	N
3†	71	CS	2	ilium	>10	IIIB	SR PI	ext	P1-4	N	N
4	72	CS	2	prox thigh	43	IIIB	PR EXULC	ext	P2/3	1	Y
5†	69	CS	3	ilium	22x18x16	IIB	PR	ext	P1-4	3	Y 68
6	65	CS	2	ilium	10x13	IIB	PR	ext	P1-4	0	N
7	69	CS	3	acetabular	6,5x10x12	IIB	PR	int extra*	P1-4	11	Y 93
8	74	CS	3	ilium	20x11x14	IIIB	PR	int*	P1c	2	N
9	72	CS	1	ilium	8x6x8	IB	SR PI	ext	P1-4	N	N
10	73	OS	3	prox femur	5	IIA	PR	int extra	P2/3	2	Y 95
11	78	PS	3	prox thigh	31x17x15	IIB	PR EXULC	ext	P2/3	0	N
12	67	CS	2	ilium	22x30	IIB	PR	int intra	P1-4	5	Y 170
13	71	CS	2	ilium	>20	IIB	PR	ext	P1-4	N	N
14†	76	PS	3	prox thigh	9x8x6	IIB	SR PI	ext	P2/3	1	N
15	67	CS	2	ilium	10x10x10	IIB	PR	int extra	P2/3	3	N
16	66	CS	3	acetabular	10x7x5	IIIB	SR LR	ext	P1-4	N	N
17	72	CS	3	acetabular	12	IIB	PR	int ext	P2/3	N	N
18	70	LMS	2	inguinal	19	IIB	PR	ext	P1-4	1	N
19	67	OS	3	acetabular	8,8x11,8	IIB	SR LR	ext	P1-4	3	Y 42
20	83	LMS	3	gluteal	16x8x5	IIB	SR PI	ext	P1-4	1	Y
21	66	PS	3	gluteal	12x9	IIB	PR	ext	P2/3	N	N
22	69	CS	1	ilium	5x6x7	IB	PR	int extra	P2-4	N	N
23	66	CS	2	ilium	18	IIIB	PR pall	int intra	P1-4	1	Y
24	70	FS	3	thigh	15x12x13	IIB	SR PI	ext	P1-4	3	Y 14
25	72	PS	3	prox thigh	NA	IIB	SR LR	ext	P2/3	N	N
26	71	CS	2	acetabular	7x6x10	IIB	PR	int extra	P1-4	1	N
27	70	PS	3	prox femur	20x13x12	IIIB	SR PI	ext	P2/3	N	N
28	65	CS	3	ilium	12x10x12	IIB	PR	ext	P1-4	3	Y 14
29	69	PS	3	gluteal	24x14x19	IIB	SR LR	ext	P2/3	N	N
30	71	CS	3	prox femur	12x10x7	IIIB	SR PI	ext	P1-4	0	N
31	65	TCMet	regressive	ilium	2x2x2	-	PR	int	P1c	N	N
32	73	PS	3	prox thigh	30x12x13	IIIB	PR	ext	P2/3	N	N
33	75	PS	2	gluteal	10x10x6	IB	PR	int extra*	P1-4	9	Y 10/61
34	65	CS	2	ilium	12x11x9	IB	PR	int	P1cHSPCL5	3	Y 38

*Abbreviations:* *†* = death on hospital ward, *GCT* = giant cell tumor, *LMS* = leiomyosarcoma, *CS* = chondrosarcoma, *OS* = osteosarcoma, *PS* = pleomorphic sarcoma, *FS* = fibrosarcoma, *TCMet* = thyroid cancer metastasis, *prox* = proximal, *NA* = not available, *PR* = primary resection, *SR LR* = secondary resection for local recurrence, *SR PI* = secondary resection post intralesional operation, *PR EXULC* = primary resection of exulcerating tumor, *PR* pall = primary R1 resection in palliative situation, *int intra* = internal intraarticular hemipelvectomy, *ext* = external hemipelvectomy, *int extra* = internal extraarticular hemipelvectomy, *int* = internal hemipelvectomy, * = patients with second-stage external hemipelvectomy due to infection; *P3* = ischiopubic region, *P2/3* = ischiopubic and acetabular region, *P1-4* = iliac, acetabular, ischiopubic and massa lateralis region, *P1c* = iliac and massa lateralis region, *P2-4* = acetabular, ischiopubic and massa lateralis region, *P1cHSPCL5* = iliac, hemisacrectomy and partial corporectomy of fifth lumbar vertebrae, *N* = No, *Y* = Yes.

**Table 3:** **Overview of patient characteristics, oncological outcome**

**Case**	**Metastasis at operation**	**Margin**	**Local recurr. (months)**	**Distant metastasis after first operation (months)**	**Time to death after distant metastasis (months)**	**Cause of death**	**Death at month after last major operation**	**Alive at month**	**Function**
1	N	1 I	N			-	-	60	CR
2	N	0 W	31			DOD	38	-	
3†	Y	0 NA	N			surg.	0	-	
4	Y	0 R	N			DOD	1	-	
5†	N	0 W	N	3	0	post surg.	2	-	
6	N	0 W	N			-	-	166	WC
7	N	0 W	N	3,5	0	DOD	1	-	
8	Y	0 W	N			DOD	24	-	
9	N	0 R	N			DOD	1	-	
10	N	0 R	N	3	-	-	-	64	NA
11	N	0 R	N			DOD	40	-	
12	N	0 W	N			-	-	22	WC/CR
13	N	1 I	N			-	-	80	WC/CR
14†	N	0 R	N			post surg.	1	-	
15	N	0 W	N			-	-	87	WC/CR
16	Y	0 W	N			other	82	-	
17	N	0 M	N			other	40	-	
18	N	0 M	N			other	95	-	
19	N	0 W	N			-	-	38	WC/CR
20	N	0 W	N			DOD	7	-	
21	N	0 W	N			NA	15	-	
22	N	0 W	N			-	-	57	NA
23	Y	1 I	N			DOD	12	-	
24	N	0 M	N			DOD	7	-	
25	N	0 W	N			NA	6	-	
26	N	0 M	N			other	4	-	
27	Y	0 M	N			DOD	9	-	
28	N	1 I	11	11	8	DOD	18	-	
29	N	0 W	N	41	8,5	DOD	48	-	
30	Y	0 W	35			DOD	48	-	
31	Y	0 W	N			-	-	19	WF
32	Y	0 R	N			DOD	3	-	
33	N	0 W	N			-	-	15	B/WC
34	N	0 W	N			-	-	7	WC/CR

*Abbreviations:* *†* = death on hospital ward, *Y* = Yes, *N* = No, *R* = radical, *W* = wide, *M* = marginal, *I* = intralesional, *DOD* = Dead of Disease, *surg.* = surgery, *post surg.* = post operation on ward, *other* = other cause of death, *CR* = crutches/cane, *WC* = wheelchair, *WF* = ambulating without walking aid, *B* = bed-ridden.

### Operation

A total of thirty-seven hemipelvectomies were performed in thirty-four patients. Primary tumor resection was performed in 59.4% of patients (n = 22) and secondary resections for recurrent (n = 5) or persistent (n = 7) tumor after inadequate procedures in 32.4% of patients. Internal hemipelvectomy was performed in thirteen patients. Three patients had intraarticular resections, seven patients had extraarticular resections and three patients had supraacetabular resections. The subtypes can be seen in Table 2. External hemipelvectomy was performed in twenty-one patients including all patients who underwent surgery for recurrent or persistent disease (n = 12; 32.4%). In three patients (8.8%) a conversion of internal to external hemipelvectomy was necessary due to otherwise non-manageable infection.

Nine patients received a hip transposition using an attachment tube as a means of reconstruction. In seven patients (53.8%) who underwent extraarticular resections, reconstruction was performed using a mega-prosthesis (MUTARS® (Modular Universal Tumor And Revision System) proximal femur mega-prosthesis, implantcast, Buxtehude, Germany). Six out of seven mega-prostheses had a silver coating. In three supraacetabular resections a reconstruction of the pelvic girdle was achieved using a poly-axial screw-rod system with a poly methyl methacrylate (PMMA) coating. One patient did not receive a reconstruction after a P3 resection with preservation of the hip joint.

### Oncology

In seven patients chemotherapy was administered. Twelve patients received radiotherapy of the pelvis; in six cases radiotherapy was performed before operation.

### Functional outcome

Due to a small number of surviving patients and a small number of patients included in this study, we did not perform functional outcome scores but used a descriptive classification to present functional aptitude and limitations of patients after the surgical procedure.

### Statistics

The Kaplan-Meier method was used to calculate the cumulative probability of survival using the day of operation as a starting point. Univariate analysis was carried out to investigate the influence of a particular single parameter.

### Patient consent

Before the initiation of treatment, all patients included in this study consented to the possibility of being included in a study with a retrospective design in the future and publication of their data in an anonymized fashion.

### Ethical approval

In Germany, a formal ethics approval is not deemed necessary due to the retrospective study design of this study.

## Results

### Duration of operation and blood loss

The mean duration of operation was 235.3 minutes (range 95–455 minutes). For internal hemipelvectomy the mean duration of operation was comparatively longer with 302.2 minutes (range 179–455 minutes) compared to 189.6 minutes (range 95–400 minutes) in external hemipelvectomy. Blood loss had to be substituted with a mean of 10.2 erythrocyte concentrates (EC) (range 2 – 42 EC) and a mean of 8.5 fresh frozen plasmas (FFP) (range 0–52 FFP). A mean blood substitution of 10 ECs (range 2 – 21 EC) was necessary in internal and 10.5 ECs (range 3 – 42 EC) in external hemipelvectomy.

### Length of Hospital Stay (LOHS)

The mean LOHS was 52 days (range 13–114 days) both for internal and external hemipelvectomy. Separated for internal and external hemipelvectomy the mean LOHS was 66 days (range 13–114 days) in internal and 43 days (range 15–91 days) for external hemipelvectomy respectively. Patients who underwent a conversion from internal to external hemipelvectomy had a mean LOHS of 70 days (range 40–114 days).

The recommended bed rest during LOHS was a mean of 15 days (range 3–70 days), with a mean of 29 days (range 5–42 days) for internal and a mean of 6 days (range 3–14 days) for external hemipelvectomy.

Three patients died on the hospital ward (patients are marked with a † in the column “Case” in Tables 2 and 3). One patient died of mass bleeding and coagulopathy on the day of operation; two patients died due to complications on the hospital ward 31 and 84 days after operation.

### Complications

Intraoperatively, one epidural bleeding and three injuries of the urogenitary system occurred. The only severe complication was mass bleeding and coagulopathy, occurring in three patients.

The most common complication observed was wound healing deficiency and infection (n = 21 (61.7%)). Surgical intervention because of infection was necessary in eighteen patients. A mean of three surgeries was necessary before secondary wound closure (range 1–11). Vacuum-assisted closure (V.A.C.®) therapy was used in twelve patients (66.7%). The mean duration of V.A.C.® therapy was 50.5 days (range 10–95 days). 69% of patients (n = 9/13) who underwent internal hemipelvectomy needed revision procedures compared to 43% in the external hemipelvectomy group (n = 9/21). Five out of seven patients underwent revision procedures in the patient collective who received a mega-prosthetic reconstruction after extraarticular resections. Two of these patients developed a deep implant-associated infection necessitating a second-stage external hemipelvectomy. All patients who underwent a second-stage external hemipelvectomy are marked using a * in the column “Type of Procedure” in Table 2. Conservative treatment with antibiotics was possible in three patients only who had undergone external hemipelvectomy.

Other procedure related complications observed on the hospital ward in decreasing order were as follows: deep vein thrombosis/pulmonary embolism (n = 5), paralytic ileus/subileus (n = 2), clostridium difficile enterocolitis (n = 1), gall bladder hydrops (n = 1), multi-organ failure (MOF) (n = 1) and temporary severe disorientation/confusion in need of treatment (n = 1). Parameters such as surgical treatment (external/internal) (p = 0.905), BMI (p = 0.677), duration of operation (p = 0.931), tumor size (p = 0.832) or radiotherapy (p = 0.107) did not influence the onset of wound healing disorders significantly.

### Oncological outcome

Resection margins were clear (R0) in 88% of patients (n = 30). Six (17.6%) radical, eighteen (52.9%) wide and five (14.7%) marginal resection margins were observed. In one case the resection margin was not available.

6% (n = 2) of patients had an accidental intralesional resection (R1), one of which was treated by adjuvant radiotherapy (high-grade chondrosarcoma) and the other by surgical re-resection (intermediate-grade chondrosarcoma).

6% (n = 2) of patients had planned intralesional resections (R1). One patient had a R1 resection for pain relief to ensure limb salvage in a highly palliative setting and didn’t undergo any adjuvant therapy (intermediate-grade chondrosarcoma with disseminated pulmonary metastases). The other patient had an intralesional resection of a giant cell tumor of bone.

Local recurrences occurred in 8.8% of patients (n = 3) at a mean time of 26 months after operation (range 11–35 months). All local recurrences occurred after external hemipelvectomies. Two local recurrences occurred in patients who had received hemipelvectomy after primarily intralesional resections (n = 1) or in recurrent disease (n = 1). Only one local recurrence occurred in a patient who had a primary resection after an external P1-4 hemipelvectomy.

Adjuvant radiotherapy (n = 1) in combination with intralesional debulking (n = 1) was administered in two patients. One moribund patient didn’t receive any adjuvant treatment.

Neither chemotherapy (p = 0.84) nor radiotherapy (p = 0.378) effected the overall survival significantly.

### Metastasis

At the time of operation four patients had solitary pulmonary metastases, three patients had bony metastases, and one patient had pulmonary and lymphatic or pulmonary, bony and soft tissue metastases each (total number of patients with distant metastases at time of operation n = 9 (26.5%)).

Five patients developed distant metastases after a mean of 13 months after operation (range 3–41 months). Pulmonary metastases occurred in four patients, bony metastases in one patient. Four patients died after a mean time of 4 months (range 0–8.5 months). One patient (high-grade osteosarcoma) achieved a complete remission with adjuvant chemotherapy and currently shows no evidence of disease (at 64 months after operation).

### Survival

At a current follow-up of a mean of 56 months (range 7 – 166 months) 35.5% (n = 11/31) of patients are alive with no evidence of disease (NED). 64.5% of patients died of disease (n = 14/31; 45.2%) or other causes (n = 6/31; 19.3%) at a mean time of 22 months after operation (range 0–95 months).

**Figure 1:**
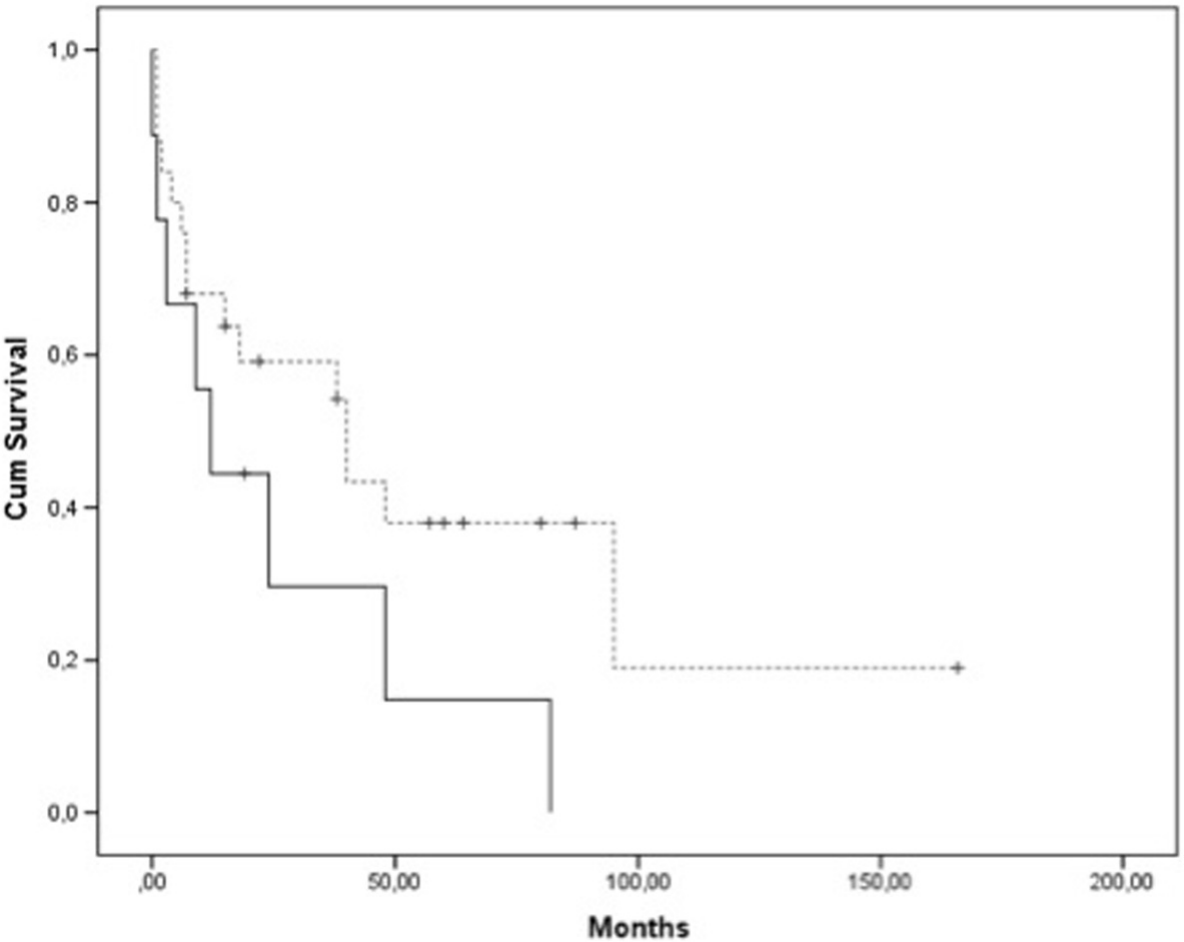
**Cumulative survival of patients (n = 9) with metastases at time of operation compared to patients (n = 25) without metastases at time of operation.**

According to Kaplan-Meier analysis, we found that palliative patients (n = 9) who presented with distant metastases at the time of operation had an estimated mean survival of 26 months (std. error 10.558; CI: 4.89-46.28) after operation compared to 61 months (std. error 15. 276; CI: 31.06-90.94) in patients (n = 25) who didn’t have metastases and were treated with a curative intent. Interestingly, there was no statistically significant influence of the event metastasis at time of operation versus no metastasis at time of operation on the overall survival (p = 0.119). However, all but one patient presenting with metastases at time of operation died of disease after an average of 22 months (range 0–82 months). The one surviving patient (Pat. 31, Tables 2 and 3) had a solitary bony metastasis of thyroid gland carcinoma and is free of disease at last follow up 19 months after operation. Patients without metastases at the time of operation died of disease after an average of 37 months after operation (Figure 1).

Significant influencing factors on survival were tumor size larger than 10 cm (p > 0.058) and the event local relapse (p = 0.027).

### Functional outcome

In eleven surviving patients, the functional outcome after operation was evaluated. After internal hemipelvectomy one patient walks freely without aids, another one uses a cane; three patients are reliant on crutches for short and a wheelchair for long distances. After external hemipelvectomy two patients are wheelchair-bound; one of them can only use it for short periods at a time and two patients rely on crutches for short and a wheelchair for longer distances. Two patients were lost to functional follow-up.

## Discussion

The results presented in this study emphasize that both internal and external hemipelvectomy are possible in elderly patients with a curative and palliative intention.

In this study, duration of operation was an average of five hours for internal and 3 hours for external hemipelvectomy. The perioperative blood transfusion rate varies greatly depending on type and extent of procedure performed. These findings are comparable with the results presented by Grimer et al. and Senchenkov et al. [5,9]. They report a mean blood loss of two liters up to an average use of 13.4 units. There is a wide range regarding published operation times though our results are in accordance with the operating times published by Grimer et al. [9].

However, we observed an increased overall surgical risk and a higher perioperative complication rate in this study population compared to younger patient collectives. In this series, we found an observed hospital mortality of 8.8% (n = 3). Other series report a mortality rate ranging from 0% to 5% [2,4,5,9]. Mass bleeding with consecutive coagulopathy and multi-organ failure has been observed as a cause and is in accordance with the findings in this study [5]. The slightly elevated mortality rate in this study might be accredited to comorbidities and inferior physical coping mechanisms in elderly compared to younger patients.

Intraoperative surgical complications in this series included urogenitary injuries (n = 3; 8.8%) and are also mentioned with a frequency of 18% by Senchenkov et al. [5].

Further, Senchenkov et al. [5] also report a correlation of operative time and hemipelvectomy extent as predictive factors of postoperative wound infection and flap necrosis. Compared to infection rates reported in recent literature (13 - 45%) our findings of 61.7% are elevated [4,5,7,9,14]. Hillmann et al. [14] published 50% of infections in patients who underwent prosthetic and allograft reconstruction or a combination of both, signifying a higher risk of infection in the presence of foreign material bodies in the wound cavity. In this study, nine of thirteen patients (69.2%) who underwent internal hemipelvectomy needed surgical wound revisions for infection, leading to external hemipelvectomy in two extraarticular (mega prosthesis reconstruction) and one supraacetabular resection (8.8%; n = 3). Reports of second-stage external hemipelvectomies for infection after a limb salvage approach have been reported in other series and compare with our findings [14]. Of twenty-one external hemipelvectomies in this study, twelve patients (57.1%) developed wound-healing disorders necessitating surgical approaches in nine patients (42.9%). Considering that operation time in this study is relatively short compared to the range published by Senchenkov et al. [5] other factors that might have caused higher infection rates among our patients have to be considered. Other reported predicting factors for wound infection such as age, immunosuppression, obesity, malnutrition, diabetes, anemia, radiation and duration of operation [5] - among others - indicate that the cause for the elevated infection rate in this study is most probably multifactorial in elderly patients, considering the fact that 47% suffered of at least one significant comorbidity in this study.

Additionally, the tumor size in this study (81.8% ≥ 10 cm) exceeds reported sizes in other collectives (35%-77.6% ≥10 cm) and might have caused both a higher rate of ablative procedures performed and wound infections [3,4,8,15].

With regard to the oncological outcome no parameter in disfavor of hemipelvectomy in patients over 65 years was found. Overall local recurrence occurred in three patients (8.8%), two of which had received a hemipelvectomy for persistent or recurrent disease. Other studies report similar and higher local recurrence rates (6-50%), indicating that hemipelvectomy in elderly patients and large tumors works as effectively for local control as it does in other collectives [2-4,7,15,16].

An overall survival of 35.5% at a current follow-up of 56 months presented in this study is comparable with other findings in literature (27%-45% at five years) [3,9,15]. The poor prognosis observed for pelvic high-grade sarcomas in literature reflects in these results as well [3,4,15,16]. Interestingly, even patients with pulmonary metastases at the time of operation presented a mean survival period of 22 months from operation to DOD (Dead of Disease). Therefore, we believe that for reasons of local control, pain relief and preservation of quality-of-life for a period of time close to two years, performing hemipelvectomy seems justified even in palliative situations.

Since most elderly patients who underwent hemipelvectomy in this study are permanently disabled regardless of the procedure performed, one has to carefully balance the advantage of preserving the patients’ physical integrity performing an internal hemipelvectomy with the disadvantages of this procedure (higher risk of complications/wound infections with secondary amputations/longer hospital stay) compared to an external hemipelvectomy especially in palliative situations. Grimer et al. [9] presented good overall results for patients with external hemipelvectomy treated with a palliative intent in terms of pain relief, improved mobilization and preservation of as much quality-of-life as possible as major goals. This study shows that external hemipelvectomy is superior to internal hemipelvectomy in terms of shorter hospitalization periods and fewer severe complications in elderly patients. Further, as Grimer et al. [9] reported we have also made promising experiences using vacuum-assisted closure devices in wound-healing deficiencies. We also continue to do V.A.C.® therapy in an outpatient clinic setting, thus reducing hospitalization periods and enabling the patients to spend more time in their domestic environment.

With regard to our results, we consider external hemipelvectomy as a feasible treatment option in elderly patients more often than in younger patient collective.

## Conclusions

Little information analyzing the surgical and oncological outcome in patients undergoing hemipelvectomy at 65 years of age or older is available in literature. We are aware of the limited validity of our results due to a small number of included cases and retrospective study design. But the findings of this case series suggest the feasibility of internal and external hemipelvectomy in elderly patients and in light of the demographic aging of our society, as well as improved oncological treatment options, we believe this issue is becoming more and more important and our results can offer a basis for the difficult treatment decisions in these patients.

## Abbreviations

MRI: Magnet resonance imaging
CT: Computed tomography
MUTARS®: Modular Universal Tumor and Revision System
EC: Erythrocyte Concentrate
FFP: Fresh Frozen Plasma
LOHS: Length of Hospital Stay
V.A.C.®: Vacuum-assisted closure
DOD: Dead of Disease
